# Effects of loop type and target direction variation on kinematics and kinetics of table tennis backhand loop: a statistical parametric mapping approach

**DOI:** 10.3389/fbioe.2025.1722210

**Published:** 2025-12-11

**Authors:** Zheng’ao Li, Liangsen Wang, Ling Zhang, Ying Hou, Yuliang Sun

**Affiliations:** School of Physical Education, Shaanxi Normal University, Xi’an, China

**Keywords:** biomechanics, backhand loop technique, lower limb, joint torque, joint angle

## Abstract

**Background:**

Modern table tennis increasingly relies on the backhand loop as a primary scoring stroke, but it remains unclear whether commonly used tactical variants—Fast-Loop versus Slow-Loop and Long-Line versus Cross-Court—place different demands on lower-limb kinetics and performance-related kinematics. This study aimed to analyse differences in backhand loop against backspin techniques among elite male table tennis players, focusing on various loop types and variations in target direction.

**Methods:**

Fifteen elite table tennis athletes performed the backhand loop. Kinematics and kinetics variables were collected synchronously. The racket velocity, joint angles of the upper and lower limbs, and torques at the lower limb were calculated and analysed using SPM1d two-way repeated measures ANOVA among the stroke phases.

**Results:**

The hip extension torque of fast-loop was significantly higher than that of slow-loop during the early phase of the stroke (*p* < 0.001). Target direction changes of the loop were dependent on early elbow flexion (*p* = 0.015) and middle trunk rotation (*p* < 0.001) during the stroke phase. The fast-loop and cross-court of loops imposed a greater holding-side lower-limb load during the follow-through phase (*p <* 0.001, *p* = 0.032).

**Conclusion:**

Lower-limb drive strategies differed between Fast-Loop (FL) and Slow-Loop (SL) in the non-holding limb. FL emphasized hip-extension torque, whereas SL emphasized knee-extension torque. Compared with Long-Line, Cross-court relied on more trunk rotation, with no additional lower limb contribution. However, FL and CC imposed higher loads on the holding lower limb during the follow-through. Practitioners should emphasize lower-limb strength and neuromuscular control to ensure loop quality and monitor follow-through braking loads, which can increase lower-limb injury risk when excessive.

## 1 Introduction

Table tennis is a complex sport that requires athletes to possess high levels of technical, psychological, tactical, and mental abilities. The loop, as a topspin technique and the most aggressive stroke in table tennis, accounts for 53% of all stroke types (Malagoli Lanzoni et al., 2014). Current trends in table tennis (Grycan et al., 2023) indicate that, based on an analysis of techniques and tactics over the past 5 decades, the frequency of backhand loops in matches has increased significantly compared to forehand loops. Additionally, the quality of the backhand loop became a key factor in the game’s success (Sumonja, 2018; Grycan et al., 2023). This emphasises the increasing significance of the backhand loop in contemporary table tennis.

The backhand loop against backspin is a crucial technique for table tennis players to gain a competitive edge during rallies (Guo, 2021). Previous research on the backhand loop against topspin and backspin balls reveals that most of the mechanical energy obtained during the stroke is transferred through the mechanical transfer between the shoulder joint and the trunk (Iino and Kojima, 2016b). However, table tennis is a complex full-body sport that heavily relies on the lower limbs’ driving capability and core control to initiate, brake, and transmit force (Zhu et al., 2023). This energy transfer appears to be closely linked to the hip extensor muscles, which act as a bridge between the lower limbs and the trunk, such as racket-head speed in loop strokes, was closely related to hip-extension torque (Le Mansec et al., 2018; Wang et al., 2018; Iino, 2018). However, most prior research on lower extremity kinetics has focused on forehand loops (He et al., 2022; Zhang et al., 2024; Yang et al., 2025). A portion of previous research on the backhand loop has focused on the kinematics and electromyography (EMG) of the upper extremity, trunk, and lower limb (Iino and Kojima, 2016a; 2016b; Wang et al., 2018; Le Mansec et al., 2018; Bańkosz and Winiarski, 2018). There has been little research on lower extremity kinetics. Thus, exploring the biomechanical changes in the lower limbs can provide practitioners with a deeper understanding of the backhand loop technique.

Given the complexity of matches, players modulated loop spin and selected target direction to ensure return quality. Typically, loops are categorised into Fast-Loop (FL) and Slow-Loop (SL). Slow-Loop, characterised by a high radius of loop (manifested as a high arc with extreme topspin), provides players with more time to prepare for the next stroke and even forces weak returns from opponents, serving as a key transitional technique to set up subsequent attacks. In contrast, Fast-Loop—defined by a flatter trajectory and maximum speed as a powerful topspin stroke—exerts significant offensive pressure on opponents and is often used as a decisive finishing shot to end rallies (Richard, 2009). Additionally, the target direction of changing the line is commonly used to create scoring opportunities in competitive table tennis (Xing et al., 2022). Previous studies on variations in return target direction have shown that changes in forehand topspin strokes are accompanied by differences in elbow flexion, racket angle, lower limb joint angles, and trunk-pelvis torques (Malagoli Lanzoni et al., 2018; He et al., 2020; 2023). Minor adjustments in stroke mechanics modulate lower-limb muscle activation and the distribution of joint torques, indicating the coupled control of the neuromuscular system (Iino, 2018; Le Mansec et al., 2018; Wang et al., 2018; Mao et al., 2023). Understanding these mechanical differences can help players and coaches better comprehend the sport and improve skill levels. However, no specific research exists on the lower limb drive that influences backhand loop target direction variation and loop type.

This study investigated the differences in joint angles and lower limb joint torques between two types of backhand loops—Fast-Loop (FL) and Slow-Loop (SL)—and two return trajectories—Long-Line (LL) and Cross-Court (CC). The aim was to explore whether there are interaction effects or independent main effects between these technical variations. We proposed the first research hypothesis that the Fast-loop has a larger lower limb drive than the Slow-group in the non-holding side. The Cross-Court needed more trunk rotation and lower limb torque contribution than Long-Line, which was the second hypothesis of this study. Our third hypothesis was that the Fast-Loop would produce a larger increase in non-racket-side hip and knee extensor moments in Cross-Court than in Long-Line across the forward-swing phase.

## 2 Materials and methods

### 2.1 Participants

An *a priori* power analysis was performed in G*Power 3.1 (Franz Faul, Germany) (ANOVA: repeated measures, within factors; 2 × 2 within-subject design, one group, measurements = 4) to detect a medium effect (Cohen’s d = 0.4) with α = 0.05 and power (1−β) = 0.80 (James, 2019). We followed a field precedent (Li et al., 2025) and planned with a medium effect size (Cohen’s d = 0.4) in the absence of closely matched priors. Fifteen elite male table tennis athletes (Mean ± SD: age 20.67 ± 2.26 years, body mass 73.71 ± 10.27 kg, height 1.78 ± 0.05 m) were recruited. All 15 athletes were certified at or above the national First-Class level, with 12.7 ± 2.7 years of formal training. All participants used a shakehand grip and were right-handed, with no history of upper or lower limb injuries within the preceding 6 months. Before the testing, all participants were informed of the research objectives and provided written informed consent. The study protocol received approval from the Scientific Ethics Committee of Affiliation (Approval No. 202416044, October 2024) and adhered to the principles of the Declaration of Helsinki.

### 2.2 Procedure

**FIGURE 1:**
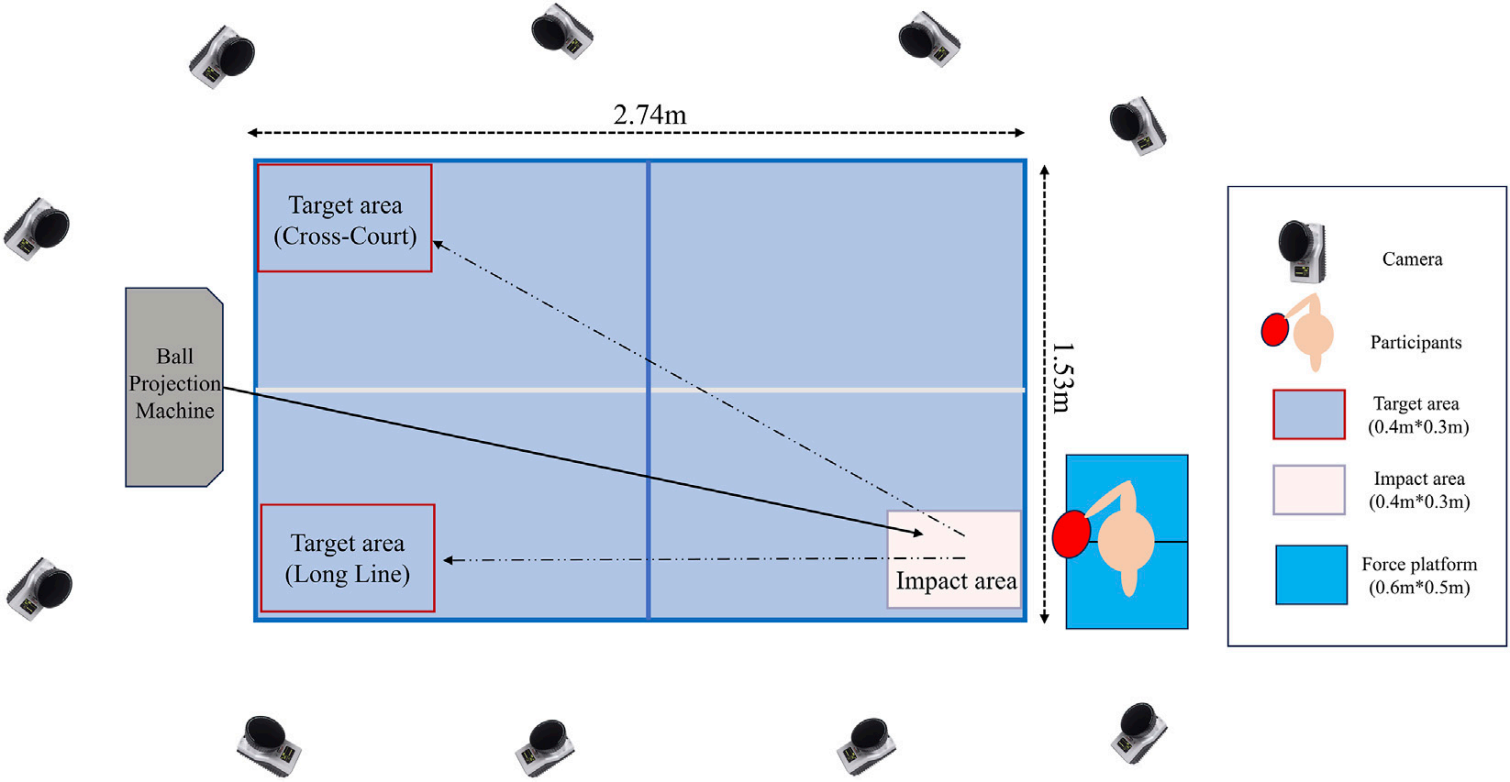
Experimental setup of the study.

The experiment was conducted in the Biomechanics Laboratory of Affiliation. The experimental setup design is illustrated in Figure 1. All participants used the same racket, which consisted of a Butterfly VISCARIA FL blade (Butterfly, Japan), with Butterfly TENERGY T05 rubber (Butterfly Technical Centre, Tokyo, Japan) on the backhand side.

A 10-camera infrared motion capture system (Oqus700+, Qualisys AB, Gothenburg, Sweden) was employed to record kinematic data at a sampling frequency of 200 Hz. Kinetic data were collected using two force platforms (Kistler 9260AA6, 0.5 m × 0.6 m; Kistler Instruments Corp., Winterthur, Switzerland) with a sampling frequency of 1,000 Hz.

All the subjects wore uniform tight pants and used a total of 70 marking points, which were set at the bony landmarks of each joint and limb of the human body, and the sticking model referred to the modified Helen Hayes model (Collins et al., 2009). A static trial was recorded for each participant in a neutral standing posture to define segment coordinate systems. A total of 3 Marker points were marked on the racket, one main central marker was located at the top of the racket, and the other two marker points were evenly distributed on both sides of the racket.

Before the formal start of the experiment, the players warmed up for 5 min (including 3 min of jogging and 2 min of dynamic muscle stretching). Then, the players were instructed to hit the backhand of the backspin ball projected by the ball projection machine (Tai De VMI 989E, Tai De, Zhongshan, China) to ensure that they were familiar with the operation process. The table tennis brand is Double Happiness Sports D40+ (D40+, Shanghai Double Happiness Sports Co., Ltd., Shanghai, China). Players then performed familiarization trials against backspin balls delivered by a ball-projection machine (Tai De VMI 989E, Tai De, Zhongshan, China). Settings were calibrated in consultation with professional coaches to approximate competition demands: frequency 30 balls·min^−1^, landing of ball preset long-ball level 2, and wheel speeds adjusted to produce stable backspin (The upper and lower rounds are set to level 2 and level 7, respectively).

During the formal test, the subjects were told that they would complete the test of four kinds of strokes of backhand loop, including the type of loop ball (FL and SL) × trajectories (LL and CC). To reduce the error, the subjects completed the test in random order. Throughout the test, the subjects should always ensure that their feet are stepped on the two force platforms respectively, and complete the backhand stroke under two different situations of stroking the ball. The stroke and target areas were standardised to 30 cm × 40 cm. Trials were deemed valid if three consecutive impacts fell within the designated landing area; the corresponding data of three shots were entered into the analysis.

### 2.3 Data analysis

**FIGURE 2:**
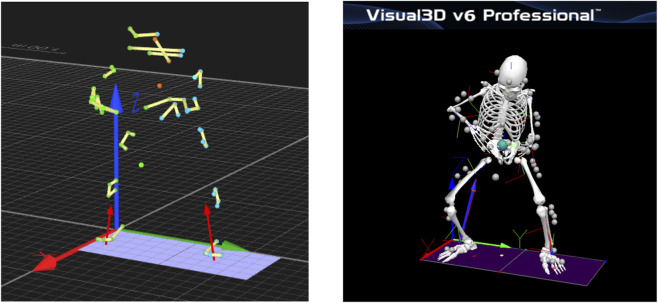
The 3D model is based on a marker set.

Preprocessed motion data (C3D format) were imported into Visual3D (v6.0, C-Motion, Germantown, MD, United States). The fourth-order Butterworth low-pass digital filter is used to filter the data, and the motion cutoff frequency and dynamic cutoff frequency are set to 14 Hz and 50 Hz, respectively (Iino and Kojima, 2016b; Mao et al., 2023). Cut-off frequencies were selected based on residual analysis and widely accepted guidelines for human-movement filtering, balancing noise attenuation with the preservation of meaningful biomechanical features (Yu et al., 1999; Winter 2009). Given that kinetic measurements from force plates contain higher-frequency components than marker-based kinematics, a higher cut-off frequency was applied to kinetics than to kinematics. Anatomic landmarks and segments are defined according to the Visual3D frame model (Figure 2) and anthropometric data. Joint angles and torques of both lower limbs and racket displacement and velocity were calculated (Cole et al., 1993; Mao et al., 2023). The pelvis is defined using the Coda model, and the angles of the ankle, hip, and knee joints are measured between the distal and proximal segments. Joint angles were determined using a Visual3D hybrid model with a Cardan X-Y-Z rotation sequence (anteroposterior, mediolateral, vertical). The joint torques were normalized by body weight (N·m/kg).

### 2.4 Definition

**FIGURE 3:**
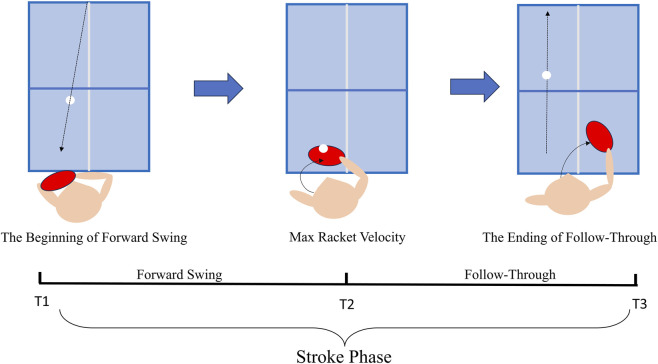
Different phases of a backhand stroke.

Based on previous studies (Bańkosz and Winiarski, 2021), the phase is divided into the backswing and stroke phases. The threshold was set to 0.1 m/s, and events were created using Visual3D. It is worth noting that the stroke phase (T1-T3) is divided into two subphases (forward swing and following the swing), which are divided by the moment of the racket’s maximum velocity (Bańkosz et al., 2020) (Figure 3). The T1 was defined as the instant when the racket’s forward-axis velocity reversed from backward to forward; T2 as the maximum resultant racket speed; and T3 as the maximum forward displacement (Bańkosz et al., 2020; Bańkosz and Winiarski, 2021).

This study focuses on analysing the biomechanical differences in the forward swing phase, standardises the forward swing and follow-through swing phase into 101 and 50 data points each (Based on the grand-mean phase-duration ratio in our sample (forward swing: follow-through ≈100%: 49.55%), and finally synthesises 150 data points for statistical analysis.

### 2.5 Statistics analysis

The one-dimensional Statistical Parametric Mapping (SPM1d) toolkit in MATLAB (R2018b, The MathWorks Inc., Beltsville, MD, United States) was used for statistical analysis. SPM1d examined the statistics for each data point within the phase, corrected using random field theory to account for quantity covariance and field smoothness (Pataky et al., 2013). Data were tested for normal distribution by using the Shapiro–Wilk test and subjected to analysis of variance (Nichols and Holmes, 2002; Mao et al., 2023). When the normality assumption was clearly violated, the corresponding effects were re-tested using the non-parametric SPM implementation of the same 2 × 2 repeated-measures model. A two-way repeated-measures ANOVA was used to compare biomechanical differences between the two loop types (factor A) and the two target direction variations (factor B), with α = 0.05 as the significance level (Pataky et al., 2015). The interaction between the two factors and the difference between factor A and factor B were discussed, respectively. When a significant interaction was observed, we conducted planned simple-effect analyses using paired-samples SPM{t} tests, with the significance level adjusted by a Bonferroni correction. Effect sizes were expressed as partial eta squared (ηp^2^) for ANOVA-type SPM{F} effects and Cohen’s dz for paired-samples SPM{t} contrasts, respectively, both computed point-wise and reported as the mean value within each significant cluster (Yang et al., 2018; Lu et al., 2024).

## 3 Results

### 3.1 Main effect for type of loop

**FIGURE 4:**
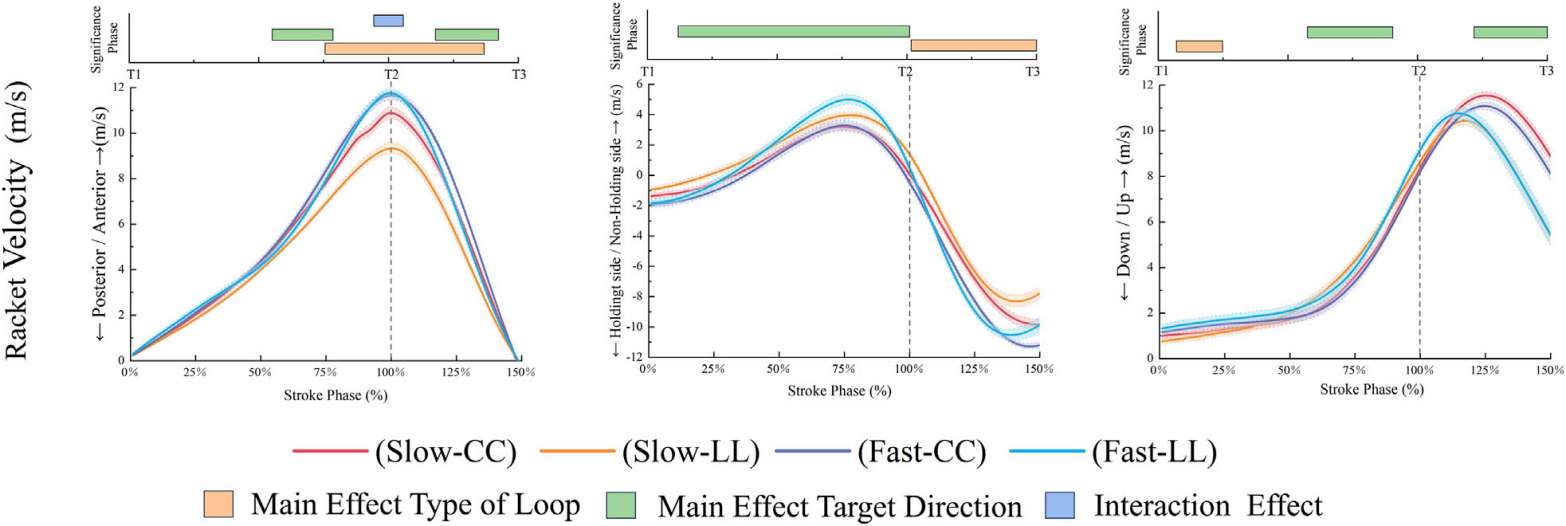
Change of racket velocity during the stroke phase.

**FIGURE 5:**
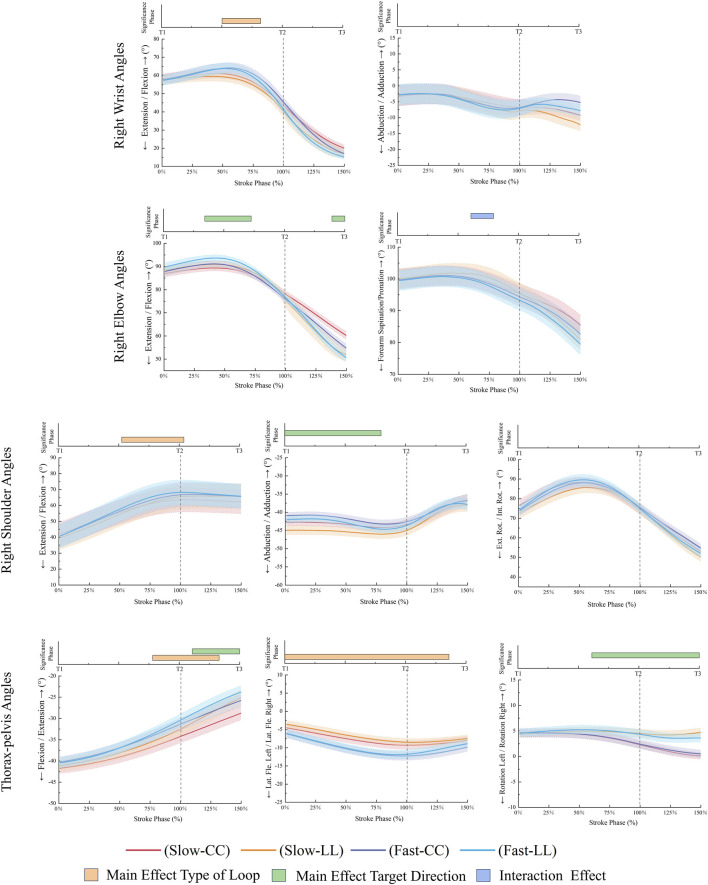
Changes of the upper limb, trunk-pelvic joint angle during the stroke phase.

**FIGURE 6:**
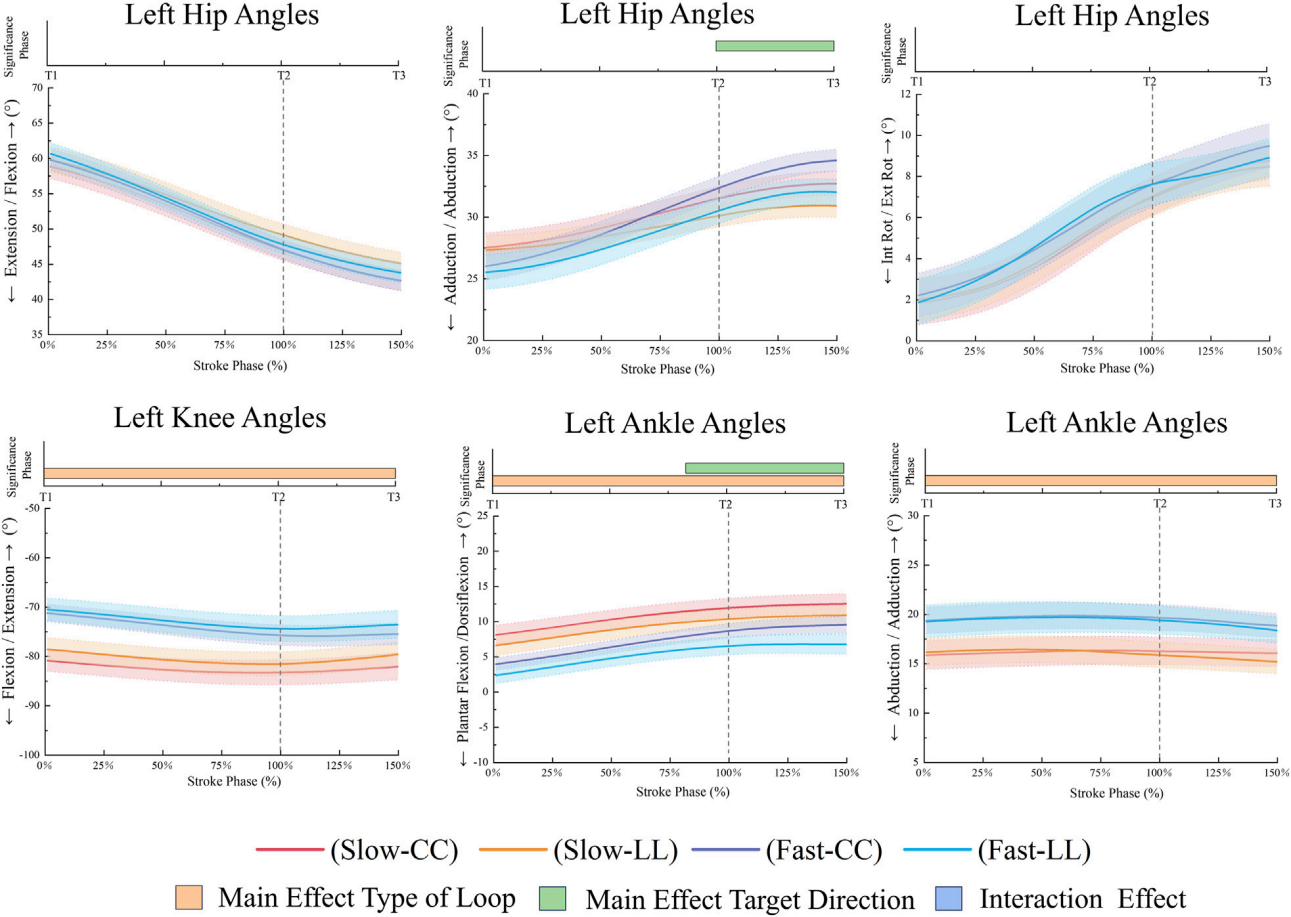
Left lower limb joint angles during the stroke phase.

As shown in Figure 4, during the stroke phase, the FL condition demonstrated significantly higher racket velocity in the anteroposterior direction compared to the SL condition (*p* < 0.001, 75.59%–136.81%, ηp^2^ = 0.52). Similarly, during the follow-through phase, the FL condition exhibited greater racket velocity (*p* < 0.001, 101.69%–150%, ηp^2^ = 0.57). In the vertical direction, the FL condition also showed higher racket velocity in the early forward swing phase (*p* = 0.011, 7.04%–24.78%, ηp^2^ = 0.41). During the forward swing phase, the FL condition resulted in greater wrist flexion (*p* = 0.021, 50.17%–81.96%, ηp^2^ = 0.46) and shoulder flexion (*p* = 0.024, 52.38%–103.86%, ηp^2^ = 0.45) compared to the SL condition (Figure 5). Conversely, the SL condition showed higher thorax-pelvis flexion (*p* = 0.014, 78.08%–133.04%, ηp^2^ = 0.36), while the FL condition exhibited greater thorax-pelvis lateral flexion (*p* < 0.001, 0%–135.88%, ηp^2^ = 0.50). The SL condition led to increased left knee flexion (*p* = 0.004, 0%–150%, ηp^2^ = 0.57) and left ankle dorsiflexion (*p* = 0.002, 0%–150%, ηp^2^ = 0.39) compared to the FL condition (Figure 6). In contrast, the FL condition showed greater left ankle adduction (*p* < 0.001, 0%–150%, ηp^2^ = 0.50).

**FIGURE 7:**
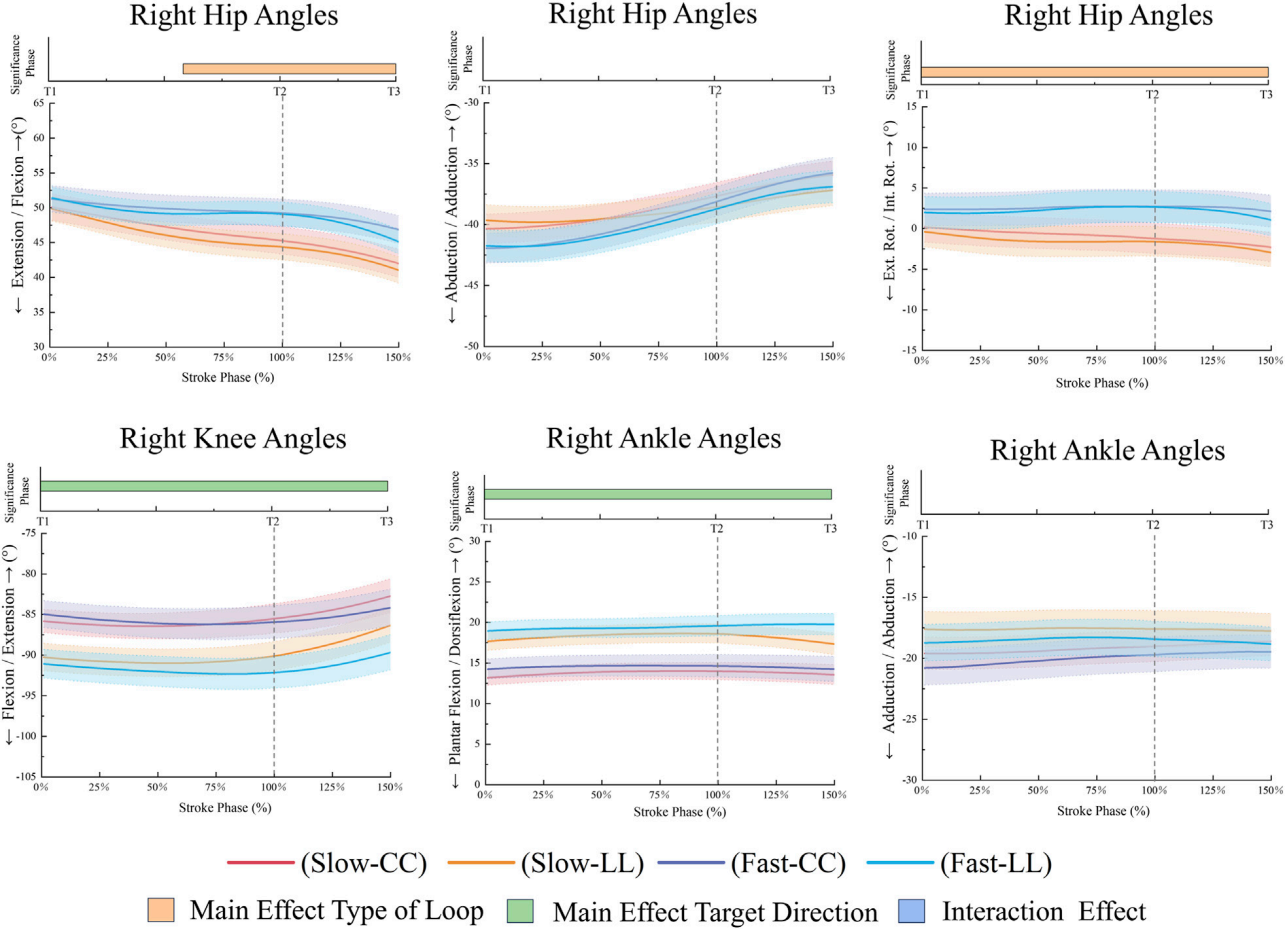
Right lower limb joint angles during the stroke phase.

**FIGURE 8:**
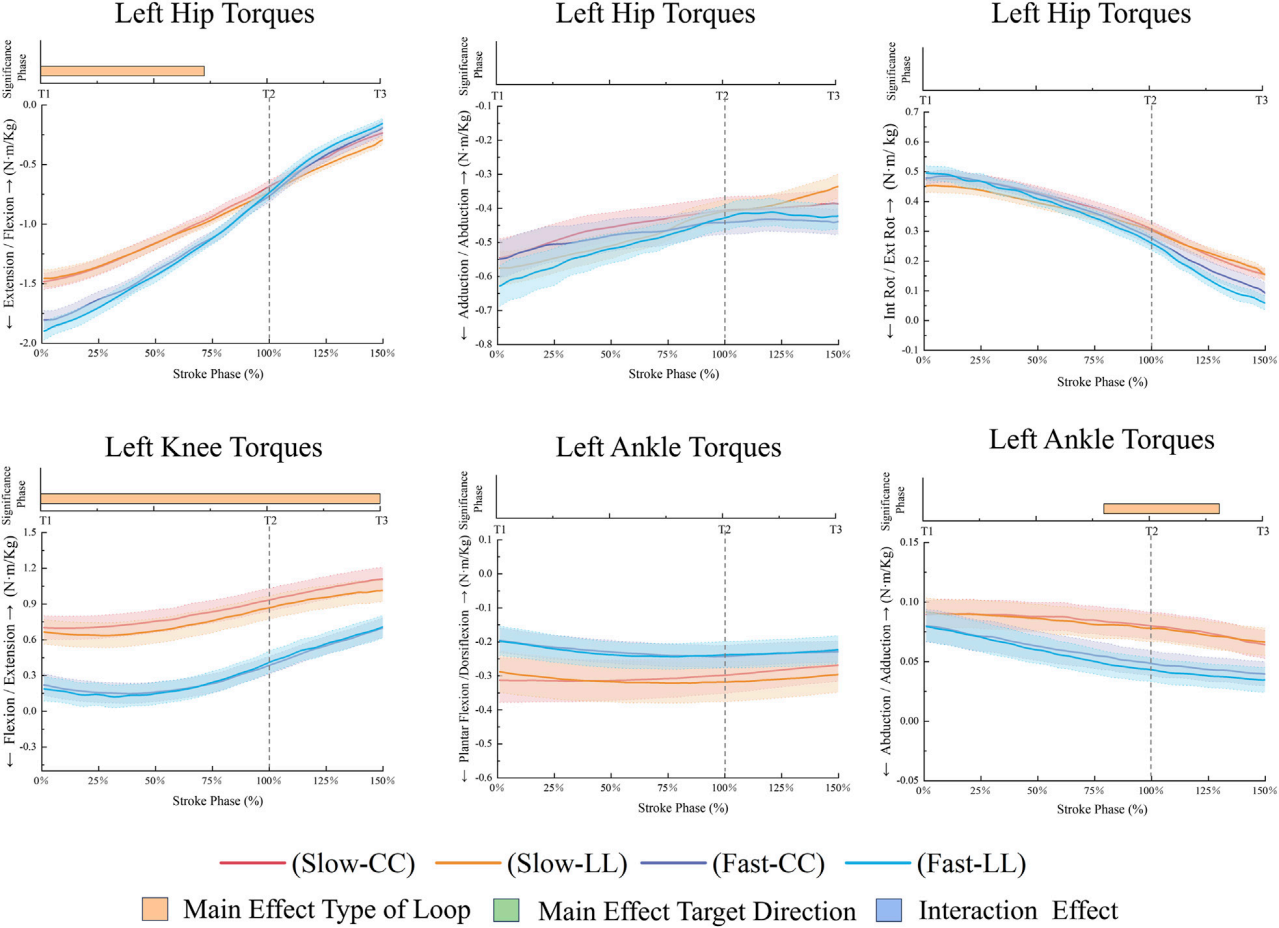
Left lower limb joint torques during the stroke phase.

**FIGURE 9:**
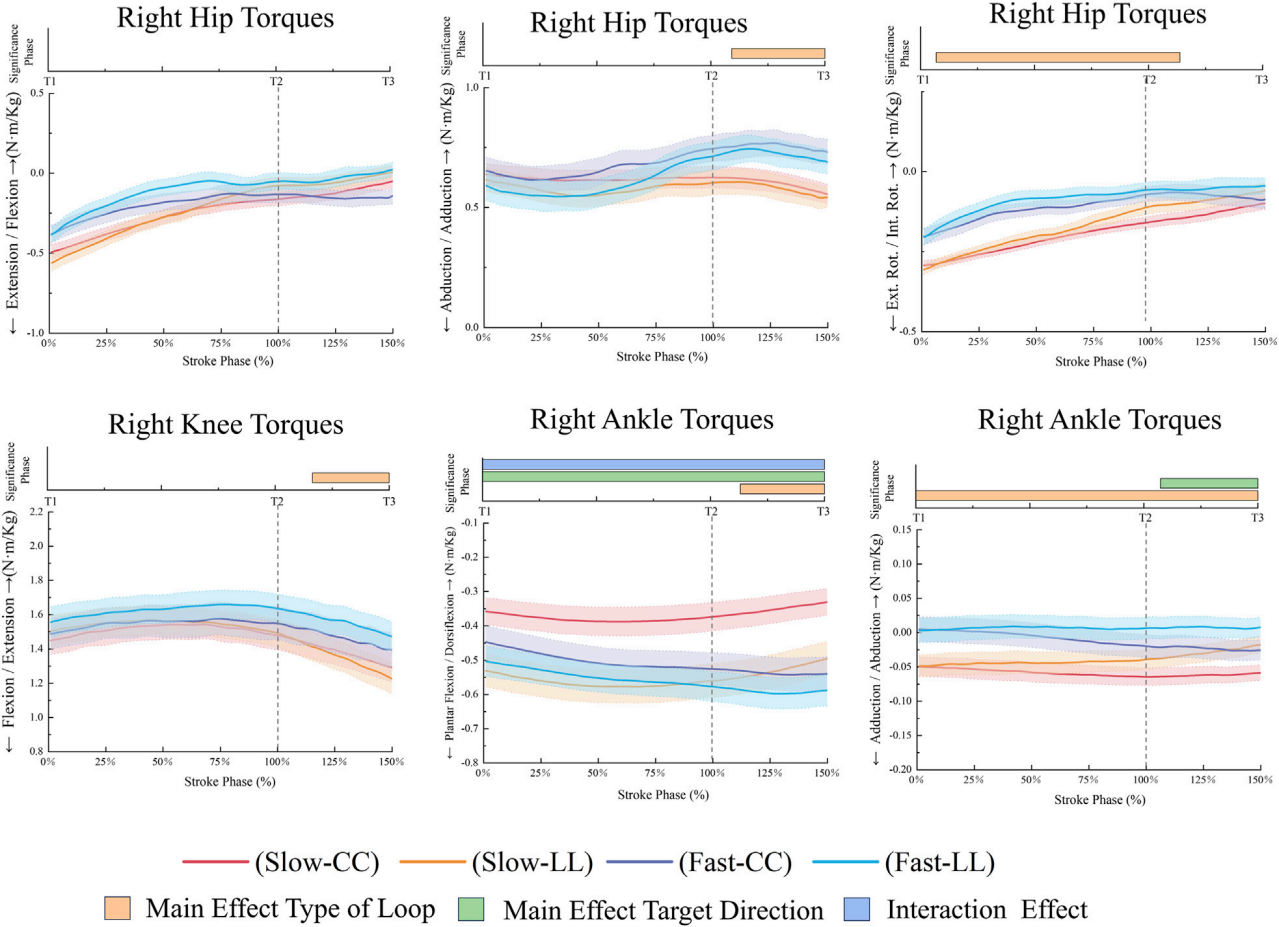
Right lower limb joint torques during the stroke phase.

The FL condition resulted in higher right hip flexion (*p* = 0.012, 58.19%–150%, ηp^2^ = 0.44) and right hip internal rotation (p < 0.001, 0%–150%, ηp^2^ = 0.58) compared to the SL condition (Figure 7). The FL condition generated higher left hip extension torques (*p* < 0.001, 0%–72.29%, ηp^2^ = 0.65), while the SL condition produced greater left knee extension torques (*p* < 0.001, 0–150, ηp^2^ = 0.72), and left ankle adduction torques in Figure 8 (*p* = 0.003, 79.92%–130.97%, ηp^2^ = 0.41). The FL condition resulted in higher right hip adduction torques (*p* < 0.001, 109.15%–150%, ηp^2^ = 0.54) and right knee extension torques (*p* < 0.001, 116.31%–150%, ηp^2^ = 0.53), while the SL condition exhibited greater right hip external rotation torques (*p* < 0.001, 0%–128.12%, ηp^2^ = 0.53) and right ankle adduction torques in Figure 9 (*p* < 0.001, 0%–150%, ηp^2^ = 0.54). Additionally, the FL condition demonstrated higher right ankle plantarflexion torques (*p* = 0.032, 113.12%–150%, ηp^2^ = 0.58).

### 3.2 Main effect for target direction variation

The distinct racket kinematics between the two kinds of factors were shown in Figure 4. The CC exhibited significantly higher racket velocity. During specific subphases of the forward swing phase (*p* < 0.001, 55.11%–78.6%, ηp^2^ = 0.47) and follow-through phase (*p* < 0.001, 118.06%–142.35%, ηp^2^ = 0.57). However, the LL exhibited significantly greater mediolateral (*p* < 0.001, 11.93%–101.04%, ηp^2^ = 0.53) and vertical (*p* < 0.001, 57.47%–90.48%, ηp^2^ = 0.46) racket velocities during the forward swing phase. Notably, this pattern reversed in the follow-through phase, with the CC achieving higher velocity values (*p* < 0.001, 121.82%–150%, ηp^2^ = 0.64). It was shown in Figure 5 that the holding side elbow displayed increased flexion during the forward swing phase (*p* = 0.015, 34.26%–72.58%, ηp^2^ = 0.48) and greater extension at follow-through termination (*p* = 0.036, 139.41%–150%, ηp^2^ = 0.41) in the LL. Concurrently, the CC condition showed enhanced shoulder abduction (*p* = 0.004, 0%–79.74%, ηp^2^ = 0.45). During the forward swing phase, the LL demonstrated larger thorax-pelvis flexion (*p* = 0.019, 110.89%–150%, ηp^2^ = 0.48) and rotation angles (*p <* 0.001, 61.13%–150%, ηp^2^ = 0.45). It was shown that CC condition participants exhibited greater left hip abduction (*p* = 0.018, 99.72%–150%, ηp^2^ = 0.46) and left ankle dorsiflexion (*p* = 0.026, 82.51%–150%, ηp^2^ = 0.51) during follow-through compared to the LL in Figure 6.

Compared with CC, LL had higher right knee flexion (*p* = 0.001, 0%–150%, ηp^2^ = 0.57) and ankle dorsiflexion angles (*p* < 0.001, 0%–150%, ηp^2^ = 0.74) were observed during the stroke phase. Figure 9 shows kinetic contrasts. The LL generated stronger right ankle extension torques during the forward swing phase (*p* < 0.001, 0%–150%, ηp^2^ = 0.39), whereas the CC displayed superior adduction torques during follow-through (*p* < 0.001, 107.29%–150%, ηp^2^ = 0.48).

### 3.3 Interaction effect

There is an interaction effect on the racket velocity in the forward and backward directions at the moment of stroke the ball (*p* = 0.002, 94.35%–105.68%, ηp^2^ = 0.47), with Slow-LL demonstrating a lower anteroposterior swing velocity (Figure 4); During the forward swing phase, Slow-LL exhibits a greater internal rotation angle of the forearm in Figure 5 (*p* = 0.023, 60.65%–79.40%, ηp^2^ = 0.46); There is an interaction effect during the stroke phase, with the right ankle of Slow-CC showing a lower plantar flexion torque (*p* = 0.001, 0%–150%, ηp^2^ = 0.44) in Figure 9.

Further *post hoc* analyses showed that, when loop type was held constant, direction-related differences in forward racket velocity were condition-specific. Within the Fast-Loop condition, no significant difference in forward racket velocity was found between Cross-Court and Long-Line strokes, whereas within the Slow-Loop condition, Long-Line strokes exhibited a lower forward racket velocity than Cross-Court strokes (*p < 0.001*, 77.47%–134.33%, *d* = 1). When the loop type was held constant, direction-dependent differences at the ankle were condition-specific. Within the Fast-Loop condition, no significant difference in racket-side ankle plantarflexion moment was observed between Cross-Court and Long-Line strokes. In contrast, within the Slow-Loop condition, Long-Line strokes produced a significantly greater racket-side ankle plantarflexion moment than Cross-Court strokes (*p* = 0.002, 0%–150%, *d* = 0.96).

## 4 Discussion

The results of this study demonstrated that changes in the type and target direction of the loop can both affect the kinematic and kinetic characteristics. Some of the results support part of the first research hypothesis, that the hip extension torque of FL is higher than that of SL, but the knee extension torque is lower than that of SL on the non-holding side. This further illustrates the difference between FL and SL in lower limb drive strategies on the non-holding side. Part of the second research hypothesis of this study was confirmed by the fact that the CC range of motion of trunk rotation was larger than that of LL. And it elicited greater racket-side ankle adduction (inversion) torque during the follow-through braking phase. However, we did not observe any additional lower-limb contribution for the Cross-Court condition during the forward-swing phase. Contrary to the third hypothesis, we did not observe a Loop Type × Target Direction interaction in the non-racket-side hip joint moment.

### 4.1 The effect of loop type (fast-loop vs. slow-loop)

It has been demonstrated that the maximum racket velocity at the moment of ball impact is a key metric for assessing the stroke quality (Mao et al., 2023), and its components in different directions were examined (Bańkosz and Winiarski, 2017). Compared with SL, FL strokes had higher peak racket velocities in the anteroposterior and mediolateral directions during the stroke phase, which results in more powerful returns and creates greater threats (2025). Previous studies have found that wrist extension, elbow extension, and shoulder joint movement have an impact on increasing the velocity of the racket in the backhand loop (Iino et al., 2008). In the present study, we found that the range of motion of the wrist and shoulder joint was larger than SL’s at the middle of the forward swing phase. Therefore, a larger flexion angle of the wrist and shoulder may produce a greater range of motion to increase the path of the racket forward swing in the stroke phase, which in turn enhances sports performance (i.e., racket velocity) during the stroke.

It was previously supposed that most of the racket’s mechanical energy comes from the trunk and lower limbs during the forward swing (Iino and Kojima, 2016b). Therefore, the velocity of the racket is closely related to the hip extension torques (Iino, 2018). This study found a more remarkable trunk lateral flexion angle and higher hip extension torque of the non-holding side in FL, which may enhance energy transfer and boost anteroposterior racket velocity. As a part of the kinetic chain of the human, the hip extensor muscle (i.e., Gluteus maximus and Biceps Femoris) generates forces that can be transmitted to the upper limbs through mechanical interaction with the spinal muscles (Vleeming et al., 1995; Le Mansec et al., 2018). In contrast, for SL, the non-holding side of knee extension and plantar flexion torques were higher than FL, possibly because the knee and ankle extensors make a greater contribution to the lower limb drive. This reflects the differences in the regulation of the neuromuscular system under the two stroke patterns. It can be presumed that the stroke pattern of FL includes large muscle groups (hip extensors) contributions, and the FL stroke likely engages large muscle groups, particularly the hip extensors, and greater hip-extension torque may be associated with higher racket velocity in the anterior direction. However, in SL (heavy-spin topspin), generating spin likely draws more on the knee-extension and ankle-plantar flexor actions, consistent with distal emphasis in the kinetic chain. Thus, we conclude that the lower limb drives of the two kinds of loops are different on the non-holding side. Moreover, it is noted that the backhand loop relies on increasing the hip and knee extension movement on the non-holding side to enhance racket velocity. However, faster racket velocity imposes a greater lower-limb load to achieve rapid braking during the follow-through phase. We found that the racket-holding side hip adduction, knee extension, and ankle plantarflexion moments were higher in FL than in SL during the follow-through phase. This braking demand warrants attention, as elevated lower-limb loading—particularly at the knee and ankle. (Biz et al., 2022; He et al., 2022). In addition, previous studies have shown that increased loading of the knee and ankle joints after fatigue may cause injury (Zhang et al., 2024; Yang et al., 2025).

Additionally, side-specific contributions may invert between forehand and backhand loops: holding-side hip extension contributes to racket velocity in the forehand (Iino, 2018), whereas the backhand may rely more on the non-holding side. Thus, the lower limb drive plays an important role in the variation of table tennis techniques, and practitioners should emphasise the importance of the lower limb drive in the stroke phase.

### 4.2 The effect of target direction variation (long-line vs. cross-court)

In the SL technique, the velocity in the anterior direction of the CC racket was higher than that of the LL. A larger racket velocity will give the ball more momentum at the moment of impact, changing the ball’s flight target direction. Previous studies have found that wrist extension and shoulder joint movement can affect the change target direction in the backhand loop (Xing et al., 2022). In this study, the elbow joint’s range of motion might affect the shot’s direction. This difference was also found when comparing the CC and LL of the forehand loop (Malagoli Lanzoni et al., 2018). However, no significant differences were found in wrist flexion, contrasting with previous research (Xing et al., 2022). This discrepancy may be due to the use of Statistical Parametric Mapping (SPM) to compare the stroke phase in this study, rather than focusing solely on the moment of ball impact. In this study, the Spm1d was used for 1D continuous data analysis, which is more complete than previous studies (peak moment of racket velocity). This method is more comprehensive than the characteristic moment and has already been applied in table tennis movements (Bańkosz and Winiarski, 2021).

Previous studies have suggested that target direction changes should focus on the trunk rotation (Malagoli Lanzoni et al., 2018). This study found that the range of motion of the trunk in the CC was larger than in the LL. Previous studies have demonstrated that the range of motion of the trunk and pelvis plays an important role in embodying greater weight transfer and greater energy production mechanisms in changing the target direction in the forehand loop technique (He et al., 2023). In the backhand loop technique that changes the target direction, a greater plantar flexion torque of the ankle joint on the holding side is exhibited in LL. It was supposed that LL needs more small muscle groups’ lower extremity contribution, and CC needs more range of motion in trunk rotation to accomplish target direction changes. The differences in target direction during backhand strokes are reflected in the control of the holding side of the distal joints and trunk rotation. It was previously supposed that the hip exertion torque of the non-playing side may play a role in controlling the racket target direction at impact in the forehand loop (Iino, 2018). Therefore, the practitioner should consider the difference between the backhand and forehand techniques in the bilateral lower limbs drive.

Moreover, during the follow-through, CC may impose a larger frontal-plane load at the ankle, reflected by greater ankle adduction moments.

### 4.3 Interaction effect (loop type vs. target direction variation)

Our study found an interaction effect for racket anteroposterior peak velocity. This was reflected in the fact that the CC peak velocity was higher than the LL in the SL, while this difference was not reflected in the FL. It is notable that the type of loop affects the change in trajectory of the loop in terms of racket anteroposterior velocity. This study also found an interaction effect of right ankle torque on the holding side during the batting phase. The LL plantarflexion torque is higher than CC in both types of loops. However, for the CC, the SL’s plantarflexion torque of the holding side is lower than that of FL’s in the stroke phase. For the backhand loop technique, SL’s non-holding side plantar drive contribution is less than FL in CC, demonstrating the difference between FL and SL lower drives.

### 4.4 Limitation

First, the players were required to keep both feet on two force platforms during the stroke, which likely restricted natural footwork and weight transfer. Lead to movement patterns that may differ from those in real match situations. Second, it was not included in the backswing phase in this research. Finally, our findings show increased lower-limb loading during the follow-through phase in the Fast-Loop and Cross-Court conditions. This study did not collect EMG data or any injury-related outcomes. Therefore, the potential injury risk associated with these loading patterns cannot be directly demonstrated. In the future, more detailed research can be conducted based on these aspects to further reveal the regular changes in the target direction and type of loop.

## 5 Conclusion

Both loop type and target direction variation significantly affected racket velocity, joint angle, and joint torque, mainly reflecting the differences in lower limb drive among different techniques. The attacking Fast-loop strokes relied on enhanced drive from the non-holding side hip extensor, characterized by significantly increased torques to enhance the force transmission of the kinetic chain. However, the higher racket velocity likely places greater follow-through load on the holding-side lower limb, increasing braking demands and raising joint moments. The Cross-Court loop was primarily completed through increased trunk rotation and elbow extension angle on the holding side compared with the Long-line loop, but it increased the ankle load in the frontal plane. These findings may support performance enhancement and help reduce injury risk, particularly where rapid braking loads are high. Future studies could further explore the neuromuscular strategies of the technique in table tennis.

## Data Availability

The raw data supporting the conclusions of this article will be made available by the authors, without undue reservation.
